# Glucose Metabolism Characteristics of Extra-Hypothalamic Cortex in Patients With Hypothalamic Hamartomas (HH) Undergoing Epilepsy Evaluation: A Retrospective Study of 16 Cases

**DOI:** 10.3389/fneur.2020.587622

**Published:** 2021-01-14

**Authors:** Yan-Feng Yang, Peng-Hu Wei, Fei Meng, Yang An, Xiao-Tong Fan, Yi-He Wang, Di Wang, Lian-Kun Ren, Yong-Zhi Shan, Guo-Guang Zhao

**Affiliations:** ^1^Department of Neurosurgery, Xuan Wu Hospital, Capital Medical University, Beijing, China; ^2^Department of Neurology, Xuan Wu Hospital, Capital Medical University, Beijing, China

**Keywords:** hypothalamic hamartomas, epilepsy, extra-hypothalamic cortex, PET, glucose metabolism

## Abstract

**Purpose:** There are few studies on the glucose metabolic characteristics of the extra-hypothalamic cortex in the hypothalamic hamartomas (HH). A comprehensive understanding of pathogenic progression of the disease is required from the perspective of cortical metabolism; therefore, we aimed to characterize metabolic characteristics of extra-hypothalamic in HH patients.

**Methods:** We investigated the metabolic characteristics of 16 HH patients, all of whom underwent epilepsy evaluation at Xuan Wu Hospital between 2017 and 2019. The lateralization and cortical distribution pattern of hypometabolism was assessed and related to HH mass neuroanatomy on magnetic resonance imaging (MRI) as well as scalp-electroencephalogram (scalp-EEG) abnormalities. Furthermore, asymmetry measurements of region of interest (ROI) in the temporal cortex (hippocampal formation, amygdala, and lateral temporal neocortex) were quantitatively assessed based on the normalized average positron emission tomography (PET) voxel values. The surgery prognosis was assessed using the International League Against Epilepsy (ILAE) classification system.

**Results:** The lateralization of hypometabolism in global visual ratings was consistent with the HH mass lateralization seen on MRI. Cortical hypometabolism showed three patterns depending whether the HH mass involved mammillary bodies, middle hypothalamus nucleus, or both. The three patterns were hypometabolism of the mesial temporal cortex with symptom of mesial temporal epilepsy (3/16, pattern I), lateral temporal, and extratemporal (frontal or parietal) cortex with symptom of neocortex temporal or frontal epilepsy (5/16, pattern II), and mesial and lateral temporal cortex and extratemporal (frontal or parietal) cortex with varied symptoms (8/16, pattern III), respectively. A significant difference in PET voxel values was found between bilateral hippocampal formation (*P* = 0.001) and lateral temporal neocortex in the third group (*P* = 0.005). We suggest that the hypometabolic characteristics of the extra-hypothalamic cortex in HH patients have three patterns. The final cortical hypometabolic pattern depends on the neuroanatomic location of the HH mass and was consistent with the main involved cortex of the interictal and ictal discharges. The third hypometabolic pattern with the most extensive cortical hypometabolism has a poorer prognosis.

## Introduction

Hypothalamic hamartomas (HH) are characterized by a variety of clinical spectrum, evolving from disease with epilepsy, central precocious puberty, or cognitive and behavioral impairment to catastrophic epileptic encephalopathy (1–6). The discovery of intrinsic epileptogenicity caused by HH has been confirmed by electrophysiology and histopathologic characterization of HH neurons (7–11). However, various surgical interventions targeting HH do not abate seizures, and the electrophysiological evidence in ineffective cases suggests that distant cortical regions are involved, especially the temporal and frontal cortex (12). This progressive epileptogenic encephalopathy suggests that the evolution of disease may correlate with abnormal cortical network external to the HH. This is consistent with the network hypothesis of focal epilepsy (13) that the epileptogenic zone is not only limited to focal lesion (HH mass) but may also involve the distant cerebral cortex external to the HH mass. Studies have confirmed that gelastic seizures are correlated with abnormal neural activation within the HH (10) and other seizure types are related to neural activation involved with cortical regions (14). These findings suggest that an independent, third-stage kindling like secondary epileptogenesis may exist in patients with HH (2, 15).

Most of the above evidence is derived from electrophysiological studies, limited by lack of spatial resolution; the lobar distribution characteristics of the cortex external to HH are not well-studied. The rapid development of imaging, functional neuroimaging techniques such as single-photon emission computed tomography (SPECT), and [^18^F]-fluorodesoxyglucose positron emission tomography [[^18^F] FDG-PET] provides additional, independent information for structural neuroimaging. The SPECT and PET tracers can measure regional cerebral blood flow and glucose metabolism associated with epileptic dysfunction. Therefore, they can be used as surrogate markers of the functional deficit cortex (16).

One SPECT study found two hyperperfusion patterns in the cortex of HH-related epilepsy (17). One pattern showed ipsilateral temporal hyperfusion and its clinical and electroencephalography (EEG) features resembled temporal lobe epilepsy. This may suggest a lateral spread pattern to the temporal lobe that determines a focal “pseudo-temporal” epilepsy. The other pattern showed ipsilateral frontal hyperfusion, and its clinical and EEG showed symptomatic generalized epilepsy with a more catastrophic syndrome. It was possible that a vertical spread pattern involved deep midline structure, such as the medial frontal cortex. Patients with catastrophic syndrome are also often accompanied by a decline in cognitive function (18, 19). Wagner et al. (6) showed in the study that regions of reduced glucose metabolism in cognitively impaired patients mainly were in the frontal and parietal cortex.

PET studies show higher sensitivity and specificity in the localization of the functional deficit cortex than SPECT studies (20). In a PET study of HH, the hypometabolic extrahypothalamic cortex, like that of electroclinical findings, varied greatly between patients and was related to most seizure types except gelastic seizure (21). The hypometabolic extra-hypothalamic cortex includes the temporal, frontal, and parietal cortex. In addition, Parvizi et al. (22) showed that the specific anatomical location of the HH mass in patients may determine the possible involvement of specific hypothalamic nuclei and neuroanatomical routes of seizure propagation in these cases. Studies showed 75% of patients with other seizure types more commonly develop focal seizures with frontal or temporal onset (23, 24). This may depend on the tight connections of HH through mammillary bodies (temporal lobe) or to the middle hypothalamic nucleus (frontal lobe) (25). It may suggest that there are hypothalamic candidate nuclei in the seizure propagation in patients with HH. HH mass may make use of the adjacent normal hypothalamic nuclei for propagation of epileptic discharges and form their own abnormal connections with extra-hypothalamic structure leading to cortical hypometabolism (22). However, little research has focused on the cortical distribution characteristics of glucose metabolism of extra-hypothalamic cortex. In this paper, we aim to investigate the abnormality of extra-hypothalamic cortex from the perspective of cortical metabolism in patients with HH and further to find whether the cortical distribution characteristics are correlated with the neuroanatomical location of HH mass and scalp-EEG findings.

## Methods

### Patients

The present retrospective study included 16 patients (nine males, seven females) with medically intractable epilepsy due to HH, all of whom underwent epilepsy evaluation, and thermocoagulation was performed at Xuan Wu Hospital between 2017 and 2019. We excluded cases with (a) previous history of surgical intervention and (b) any brain abnormalities other than HH in image. [^18^F] FDG-PET scans were acquired in patients with multiple seizure types other than gelastic seizure or with severe cognitive or behavioral disorders, completely in accordance with clinical needs. In addition, EEG and MRI were acquired in the context of presurgical evaluation and were available for retrospective analysis. The clinical characteristics of the included patients are shown in Table 1. The mean age of patients was 11.75 ± 10.31 years (range 2–37 years). The mean age at seizure onset was 3.73 ± 4.08 (range 0.08–16 years). The study was approved by the ethics committee of the Capital Medical University, and all included patients and caregivers provided written informed consent to participate and for publication.

**Table 1:** Demographic and clinical features of patients (*n* = 16).

**No**.	**Sex/age (years)**	**Age (years)**	**Onset age (years/months)**	**Classification**	**Seizure types**	**Other manifestation**	**ILAE class/outcome (months)**
1	M	18	16 years	II	GS (1–2c/m); FGTCS (1–2c/y)	None	1 (8)
2	F	7	6 years	III	GS (1–3c/d); FIAS (1c/3–4d)	PP	1 (6)
3	M	37	1 years	III	GS (1–2c/m); FIAS (1–2c/m)	None	1 (21)
4	M	18	3 months	I	GS (2–3c/d); FIAS (2–3c/m)	BD	1 (10)
5	F	6	4 months	III	GS (10c/d); FIAS (1–2c/w); FGTCS (1–2/m)	PP	4 (21)
6	F	7	6 months	IV	GS (8–10c/d); FIAS (1–2c/w); FGTCS (1–2~5–6c/w)	PP, BD	5 (9)
7	F	6	2 years	I	GS (4–5c/d); FIAS (1c/w)	PP, BD	4 (9)
8	M	3	1 months	IV	GS (20–30c/d); FIAS (3–4c/d)	BD	4 (8)
9	M	6	5 years	III	GS (4–5c/d); FIAS (1–2c/w)	None	3 (24)
10	F	7	6.5 years	I	GS (3–4c/d)	PP	1 (17)
11	M	3	3 years	II	GS (8–10c/d); FGTCS (1–2c/m)	BD	1 (10)
12	M	2	1 years	II	GS (6–10c/d); FGTCS (1c/m~3c/d)	None	4 (8)
13	M	22	2 years	II	GS (2–3c/d)	BD	1 (24)
14	M	30	3 years	II	GS (4–5c/d); FGTCS (1–2/w)	BD	4 (10)
15	F	8	6 years	III	GS (2–3c/d); FGTCS (1–2c/w)	None	1 (33)
16	F	8	7 years	III	GS (1–2c/m); FGTCS (2–3c/Y)	None	4 (17)

*ILAE classifications include the following: class 1: completely seizure free (no auras); class 2: only auras (no other seizures); class 3: 1–3 seizure-days/year; class 4: 4 seizure-days/year to a decrease of 50% from pretreatment seizure frequency; class 5: from 50% decrease up to 100% increase; class 6: ≥100% increase in seizure frequency*.

*GS, gelastic seizure; FIAS, focal impaired awareness seizure; FGTCS, focal to generalized tonic-clonic seizure; PP, precocious puberty, BD, behavioral disorder; ILAE, International League Against Epilepsy*.

### MRI Data

All patients underwent acquisition of a T1-weighted images, T1-magnetization-prepared rapid-gradient echo (MPRAGE) and T2-weighted images (Siemens 3T). The diagnosis of the hamartoma was based on MRI of the brain, which was isointense to cerebral cortex on T1-weighted images and isointense or hyperintense on T2-weighted images. The classification of HH was classified according to Delalande and Fohlen's study by two experienced imaging doctors blinded to clinical information (26). The classification is defined as horizontal base of attachment below the normal position of the floor of the third ventricle (class 1), vertical plane of attachment to the wall of the third ventricle, completely above the normal position of the floor of the third ventricle (class 2), the plane of attachment that extends both above and below the normal position of the floor of the third ventricle (class 3), and giant lesions which are bigger than 8 cm^3^ (class 4). The HH mass lateralization on MRI according to the asymmetric attachment to the hypothalamus proper with description of this by category: (1) right (right-sided and predominantly right-sided); (2) left (left-sided and predominantly left-sided); and (3) not lateralized. The hamartoma connection to the middle (middle hypothalamic nuclei) and posterior segments (mammillary bodies) of the hypothalamus was determined. Involvement of the mammillary bodies was accepted when the T1-weighted images showed there was an interruption on the continuity of the hypersignal in surrounding these structures which were in contact with the HH mass. This criterion was important because the structure of the mammillary bodies remained unchanged even if there was a significant shift in some patients (25).

### Analysis of Long-Term Scalp-EEG Monitoring and Clinical Semiology

Prolonged EEG data were acquired *via* video-EEG monitoring (Micromed, Italy). The recording parameters were as follows: sampling rate = 512 Hz, low filter setting = 0.16 Hz, and high filter setting = 70 Hz. Each EEG sample was analyzed and classified according to the following criteria: (a) lateralization: (i) left, (ii) right, or (iii) bilateral; (b) localization: (i) frontal, (ii) temporal, (iii) parietal, (iv) occipital, (v) vertex, or (vi) not applicable (NA, when generalized or non-localized); (c) abnormal waveform: (i) background slowing, (ii) spike, (iii) spike and wave complex, (iv) polyspike complex. Three independent clinicians utilized Lüders' semiological seizure classification to evaluate ictal semiology (27).

### [^18^F] FDG PET Data

#### Acquisition

PET images were acquired on a United Imaging PET scanner, providing 2.4-mm-thick slices, with an isotropic spatial resolution of 5 mm. Patients were requested to report any seizures on the day of the scan. The scans cannot be performed if patients had a seizure within the last 12 h. Patients were instructed to lie at rest in a semidark room with eyes closed and ears unplugged when a bolus of 5–10 mCi of FDG was then injected intravenously. After an uptake phase of 60 min, we performed a 30–40-min duration image acquisition. During acquisition, a facemask ensured the stable position of the head in these patients. The resulting PET images were qualitatively reviewed by an experienced nuclear physician, blinded to other clinical information.

#### Global Visual Ratings and Quantitative Analysis of [^18^F] FDG PET

The global visual ratings of the patients' lateralization and cortical distribution of hypometabolism were classified based on the asymmetrical assessment of cerebral cortex which was identified during a multidisciplinary patient management conference at the Epilepsy Center. Then the results were checked with the PET report interpreted by an experienced PET physician blinded to clinical information. The standard used to determine hypometabolism on PET reports is an asymmetrical standard uptake value (SUV) of the bilateral cortex of more than 10%. If interpretation differed, a consensus opinion was reached after joint review.

Image analysis was conducted with tools from the FMRIB Software Library (https://fsl.fmrib.ox.ac.uk/fsl/fslwiki/FslInstallation), Statistical Parametric Mapping software (SPM 12, Wellcome Department of Imaging Neuroscience, www.fil.ion.ucl.ac.uk/spm12) and 3D Slicer (http://www.slicer.org). Brain extraction tools were first used to remove non-brain tissue from structural MR images. Subsequently, T1-MRI and PET images were co-registered and spatial normalization was computed from the SPM12 T1-MRI brain template referenced by the Montreal Neurological Institute (MNI) and applied to all images. The obtained image was smoothed using 3D isotropic Gaussian kernel of 3 mm in width (28). Three regional masks were utilized to extract PET values for hippocampal formation, amygdala, and lateral temporal cortex. The masks were obtained from the FreeSurfer parcellation map and contained using FreeSurfer (http://surfer.nmr.mgh.harvard.edu). To reduce intersubject variance before performing statistical analysis, the voxel value for each patient's PET images was divided by the average voxel value measured in the cerebellum (29). Before doing so, an investigator performed a visual assessment of all images to ensure that there were no metabolic changes in the cerebellum.

In addition, metabolic asymmetric value (MAV) of the ROI of the temporal cortex (hippocampal formation, amygdala, and lateral temporal neocortex) was quantitatively assessed based on the normalized average PET voxel values. The MAV can be calculated with the following formula: MAV = (contralateral value—ipsilateral value)/ipsilateral value. We regarded normalized regional differences >10% as asymmetrical (30).

At last, we further analyzed the asymmetry value of the temporal cortex (hippocampal formation, amygdala, and lateral temporal neocortex) of the different cortical hypometabolic pattern groups based on the normalized average PET voxel value.

### Prognosis Evaluation

The surgery prognosis was assessed according to the ILAE (31) classification system through telephone calls and outpatient visits. ILAE outcome classification is an ordinal scale based on seizure-days/year, with categories defined as complete seizure freedom without auras (class 1), only auras without other seizures (class 2), 1–3 seizure-days/year (class 3), 4 seizure-days/year to a decrease of 50% from pretreatment seizure frequency (class 4), from 50% decrease up to 100% increase (class 5), and ≥100% increase in seizure frequency (class 6).

### Statistical Analysis

The normalized average PET values were expressed as median [interquartile range (IQR)] and compared between the contralateral and ipsilateral temporal cortex with the Wilcoxon signed-rank test. Wilcoxon signed-rank tests were calculated with the Statistical Package for the Social Sciences for Windows (Version 22; IBM, Armonk, New York, USA).

## Results

### Laterality of HH on MRI and Cortical Glucose Hypometabolism

HH lateralization seen on MRI was consistent with the lateralization of reduced glucose metabolism in global visual ratings. In seven of the 16 patients, HH mass was attached or predominantly attached to the left hypothalamus, and the hypometabolism of the extra-hypothalamic cortex was on the left hemisphere. In another nine patients, HH attached or predominantly attached to the right hypothalamus, causing lateralization of the cortical hypometabolism in the right. For further details, see Table 2.

**Table 2:** PET, MRI, and scalp-EEG characteristics of HH patients.

**No**.	**Lateralization**					**Localization**		
	**Hamartoma (MRI)**			**PET**	**EEG**	**PET**	**EEG**	
	**Attachment side**	**Middle hypothalamic nucleus**	**Mamillary bodies**				**Interictal**	**Ictal**
1	Rt	Yes	No	Rt	Bil, Rt. Dom	Lat_TLC, FLC	Fronto-temporal Frontal dom	R Hemisphere EDE
2	Rt	Yes	Yes	Rt	Rt	Mes_TLC, Lat_TLC, PLC	Temporal	Diffuse EDE or temporal PFA
3	Rt	Yes	Yes	Rt	Rt	Mes_TLC, FLC, PLC	Diffuse	Diffuse EDE
4	Rt	No	Yes	Rt	Rt	Mes_TLC	Temporal, sphenoid dom	Temporal PFA
5	Rt	Yes	Yes	Rt	Bil, Rt. Dom	Mes_TLC, Lat_TLC, FLC, PLC	Diffuse	Diffuse EDE or PFA
6	Rt	Yes	Yes	Rt	Bil	Mes_TLC, Lat_TLC, FLC, PLC	Diffuse	Diffuse EDE
7	Lt	No	Yes	Lt	Lt	Mes_TLC	Temporal	Temporal EDE
8	Lt	Yes	Yes	Lt	Bil	Mes_TLC, Lat_TLC, FLC, PLC	Diffuse	Diffuse EDE or PFA, fronto-central-parietal dom
9	Lt	Yes	Yes	Lt	Lt	Mes_TLC, Lat_TLC, PLC	Diffuse	Diffuse EDE
10	Lt	No	Yes	Lt	Lt	Mes_TLC	Temporal, sphenoid dom	No change or temporal EDE
11	Lt	Yes	No	Lt	Lt	Lat_TLC	Fronto-temporal	No change
12	Rt	Yes	Yes	Rt	Bil, Rt. Dom	Mes_TLC, Lat_TLC, FLC, PLC	Temporo-parietal	Temporo-parietal slow waves with spike or Rt. hemisphere EDE
13	Lt	Yes	No	Lt	Lt	Lat_TLC, PLC	Temporo-parietal	Diffuse EDE
14	Lt	Yes	No	Lt	Lt	Lat_TLC	Fronto-temporal	No change
15	Rt	Yes	No	Rt	Rt	Lat_TLC, PLC	Temporo-central-parietal	Diffuse EDE
16	Rt	Yes	Yes	Rt	Rt	Mes_TLC, FLC, PLC	Diffues	Diffuse EDE or PFA

*Bil, bilateral; Lt, left; Rt, right; Dom, dominant; Lat, lateral; Mes, mesial; TLC, temporal lobe cortex; FLC, frontal lobe cortex; PLC, parietal lobe cortex; EDE, electrodecremental events; PFA, paroxysmal fast activity*.

### Cortical Distribution Pattern of Hypometabolism and Correlation With Neuroanatomy of HH on MRI and Electroclinical Abnormalities

The extra-hypothalamic cortex in patients with HH mainly presented three hypometabolic patterns, which were related to the neuroanatomical location of HH mass and electroclinical abnormalities (see Table 2). In three patients, the HH mass established a connection only to the posterior areas of the hypothalamus (mammillary bodies), in five patients, the connection to the middle areas of the hypothalamus (middle hypothalamic nucleus), and in another eight patients, the connection to both the mammillary bodies and middle hypothalamic nucleus.

**Figure 1:**
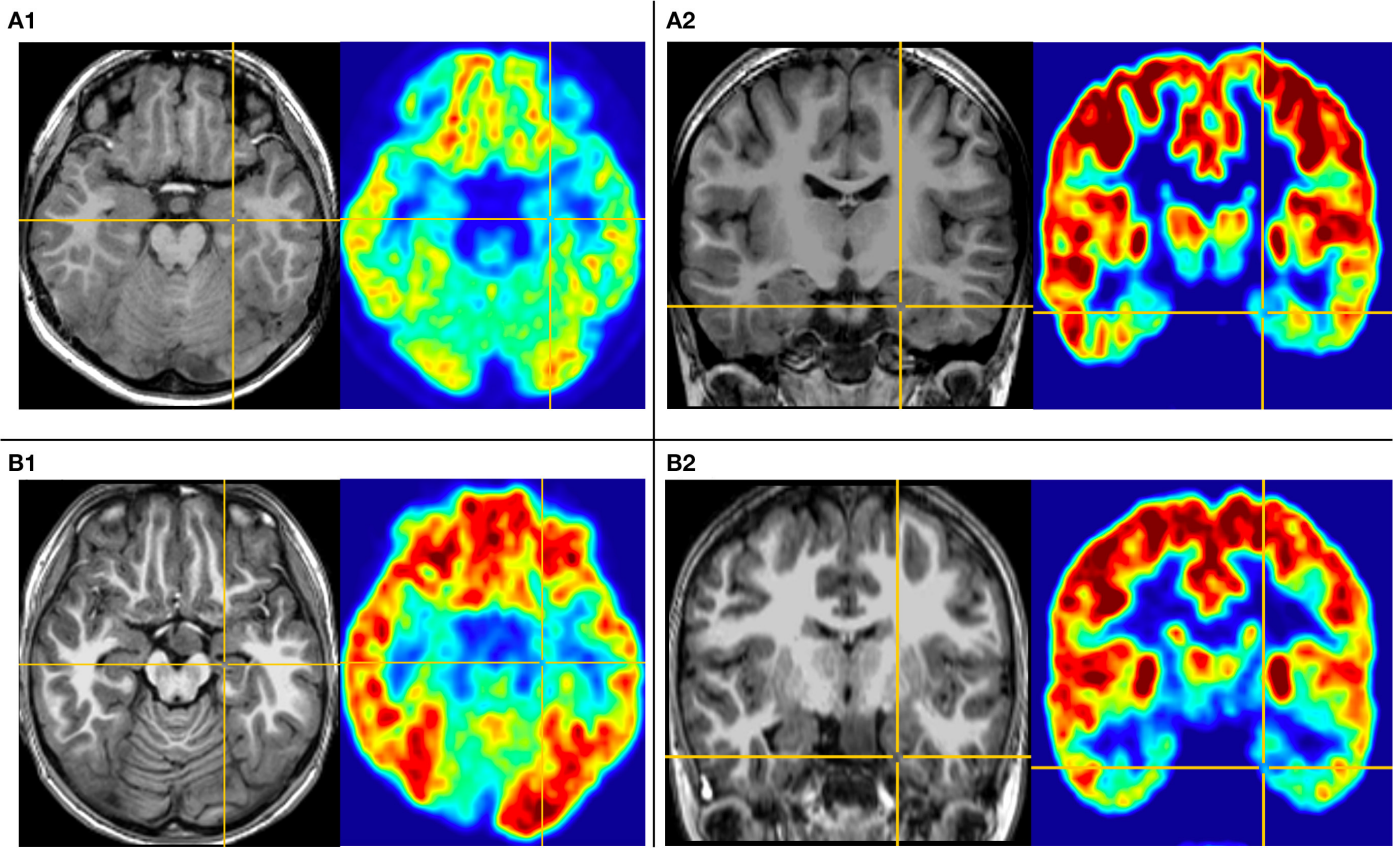
This is the MRI-PET of Pt. 10 **(A)** and Pt. 7 **(B)** in the first cortical hypometabolic pattern group. T1-MPRAGE (left) and PET images (right) were co-registered and normalized in MNI reference. **(A)** Both the horizontal **(A1)** and the coronal **(A2)** plane of PET showed that the hypometabolic cortex is located in the left mesial temporal cortex. Quantification analysis of Pt. 10 showed the PET voxel value of the right hippocampus formation of 1.970, and for the left hippocampus formation of 1.771, with an asymmetric value of 11.3%. **(B)** Both the horizontal **(B1)** and the coronal **(B2)** of PET showed that the hypometabolic cortex is located in the left mesial temporal cortex. Quantification analysis of Pt. 7 showed the PET voxel value of the right hippocampus formation of 1.962, and for the left hippocampus formation of 1.759, with an asymmetric value of 11.5%.

**Figure 2:**
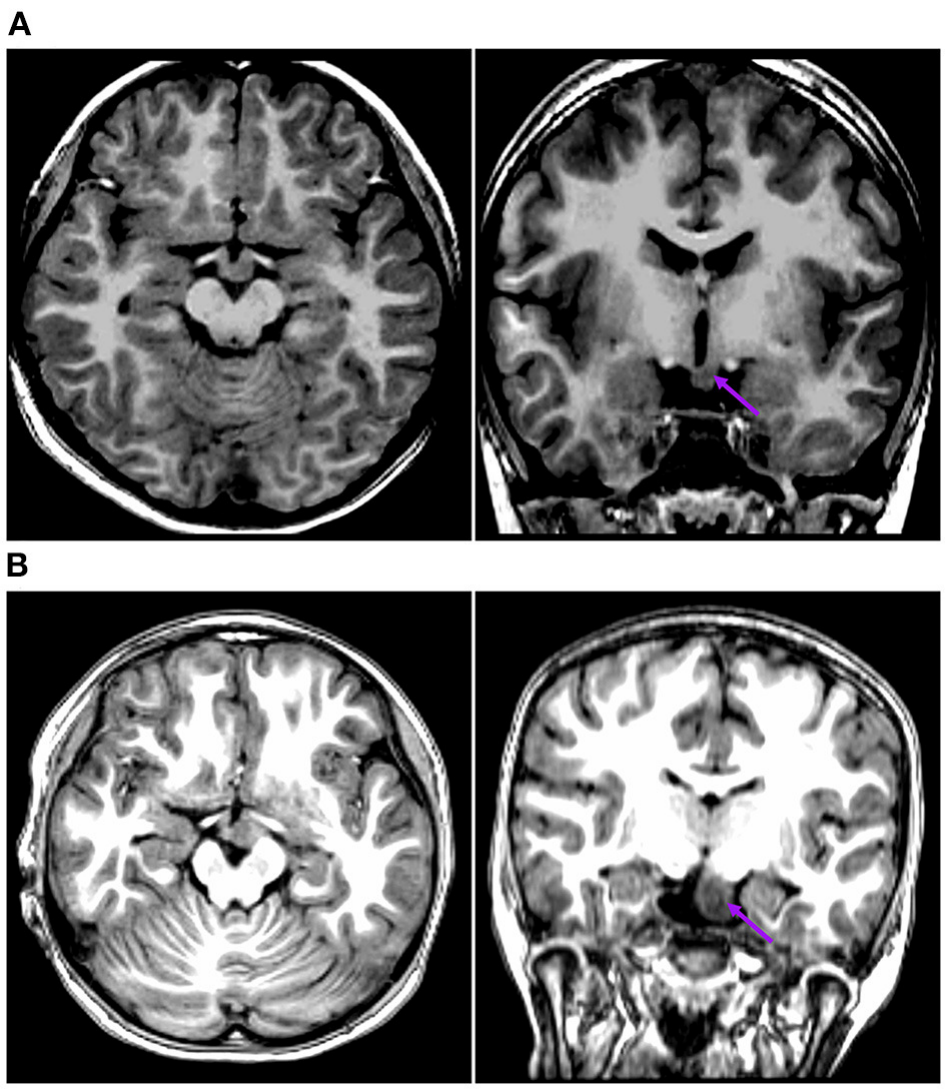
This is the raw T1-MPRAGE images of Pt. 10 **(A)** and Pt. 7 **(B)** in the first cortical hypometabolic pattern group. The T1-MPRAGE images (arrows) of **(A)** and **(B)** showed an interruption on the continuity of the hypersignal of the mammillary bodies, and the structure in contact with the HH mass was unclear. The HH mass were entirely below the floor of the third ventricle, which hardly connected to the middle nuclei of the hypothalamus.

The first pattern (pattern I) showed three patients (Pt. 4, 7, and 10) whose cortical hypometabolism was mainly in the limbic cortex of the mesial temporal lobe, including the hippocampal formation, parahippocampal gyrus, or entorhinal cortex. Three patients showed reduced glucose metabolism only in the mesial temporal cortex (Figure 1). The T1-MPRAGE of the three patients showed HH masses were entirely below the floor of the third ventricle with minimal ventricular displacement. The T1 image showed an interruption on the continuity of the hypersignal surrounding these structures in contact with the HH mass, which implied the tight connection between HH mass with the mammillary bodies (Figure 2). Of the three patients with the first hypometabolic pattern, interictal discharge predominantly occurred in the temporal cortex with the sphenoid electrode predominantly (two of three). The ictal EEG showed no change (one of three), temporal paroxysmal fast activity (one of three), or temporal electrodecremental events (two of three). The seizure types included gelastic seizure (three of three) and focal impaired awareness seizure (two of three).

**Figure 3:**
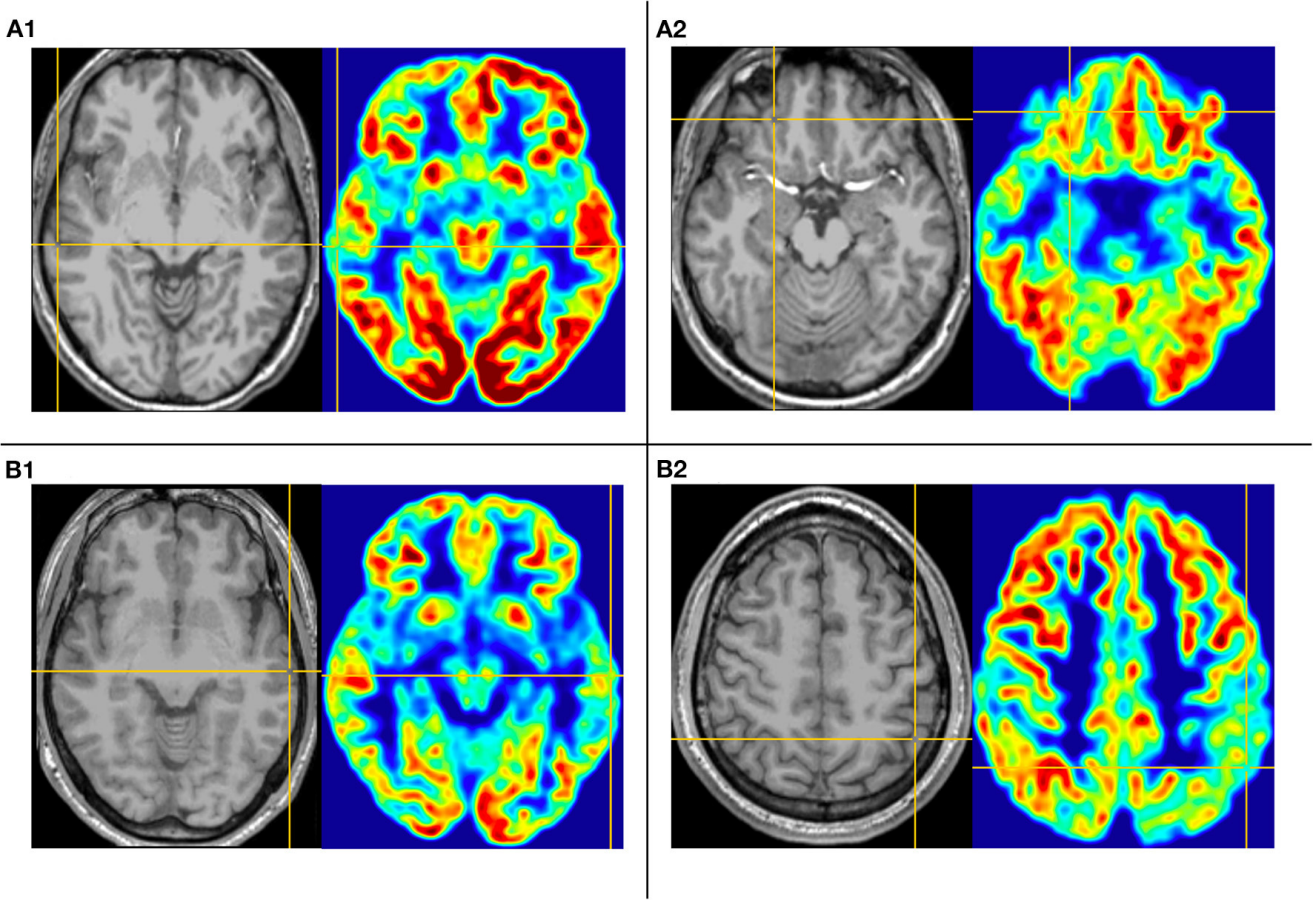
This is the MRI-PET of Pt. 1 **(A)** and Pt. 13 **(B)** in the second cortical hypometabolic group. T1-MPRAGE (left) and PET images (right) were co-registered and normalized in MNI reference. **(A)** The cortical hypometabolic region is located in right lateral temporal cortex **(A1)** and right frontal cortex **(A2)**. **(B)** The cortical hypometabolic region is located in the left lateral temporal cortex **(B1)** and left parietal cortex **(B2)**.

**Figure 4:**
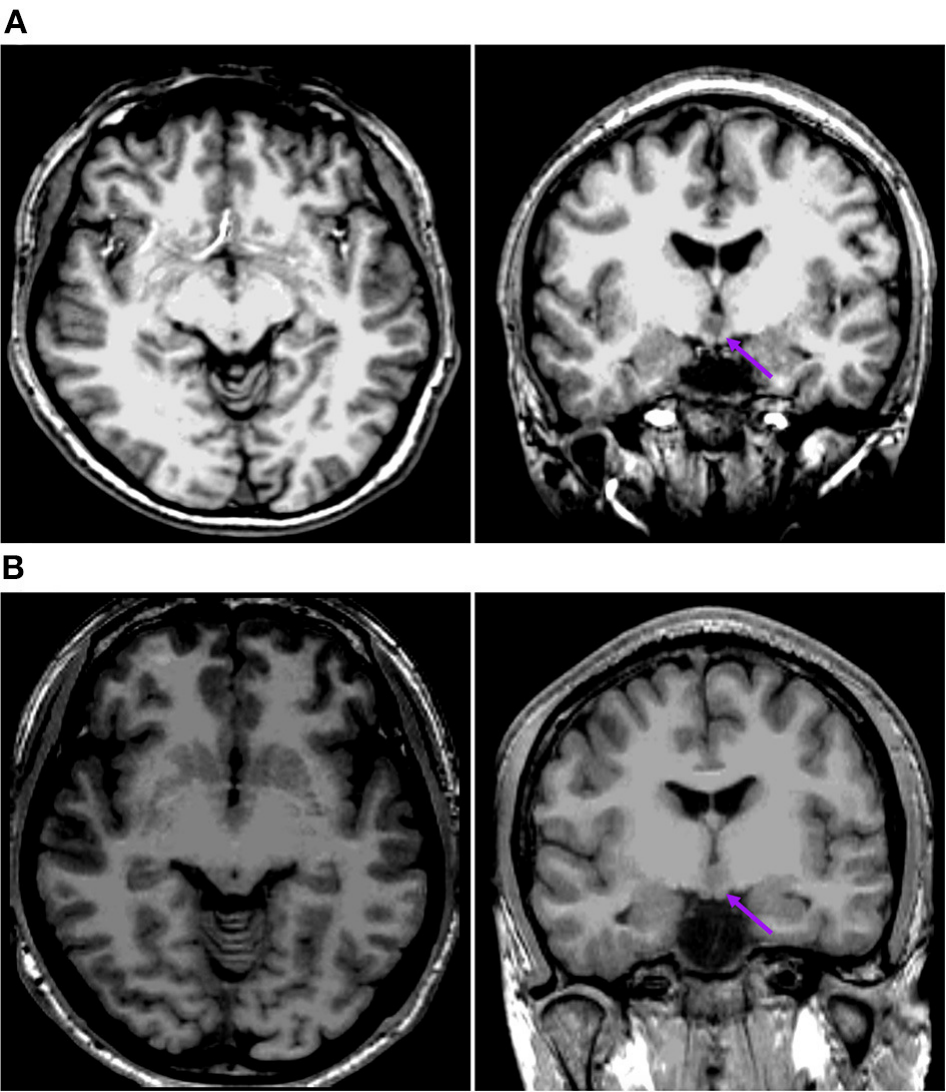
This is the raw T1-MPRAGE images of Pt. 1 **(A)** and Pt. 13 **(B)** in the second cortical hypometabolic pattern group. The T1-MPRAGE images (arrows) of **(A)** and **(B)** showed the mammillary bodies were surrounded by a ring of hypersignal with no interruption and the structure of the mammillary bodies unchanged. The HH mass were entirely above the floor of the third ventricle, tightly connected to the middle nuclei of the hypothalamus.

The second pattern (pattern II) showed five patients (Pt. 1, 11, 13, 14, and 15) whose cortical hypometabolism was mainly in the lateral temporal cortex. It may also involve the extratemporal cortex, such as the frontal cortex and the parietal cortex, while the mesial temporal cortex is rarely affected (Figure 3). The lateral temporal cortex was involved in all five patients. Of these patients, two (Pt. 11 and 14) had hypometabolism only in the lateral temporal cortex, one (Pt. 1) had hypometabolism in the lateral temporal cortex and the frontal cortex (Figure 3A), and two (Pt. 13 and 15) had hypometabolism in the lateral temporal cortex and the parietal cortex (Figure 3B). The T1-MPRAGE of these patients showed that HH masses were entirely above the floor of the third ventricle. These HH masses were located in the middle hypothalamus, sparing the mammillary bodies. There was no interruption on the continuity of the hypersignal in the T1 image, with the structure of the mammillary bodies unchanged (Figure 4). Of the five patients with the second hypometabolic pattern, the main interictal discharge occurred in the frontal-temporal area (three of five) or the temporal-parietal area (two of five). The ictal EEG showed right hemisphere paroxysmal fast activity (one of five), no change (two of five), or diffuse electrodecremental events (two of five). Seizure types in this group included gelastic seizure (five of five) and focal to generalized tonic-clonic seizure (four of five).

**Figure 5:**
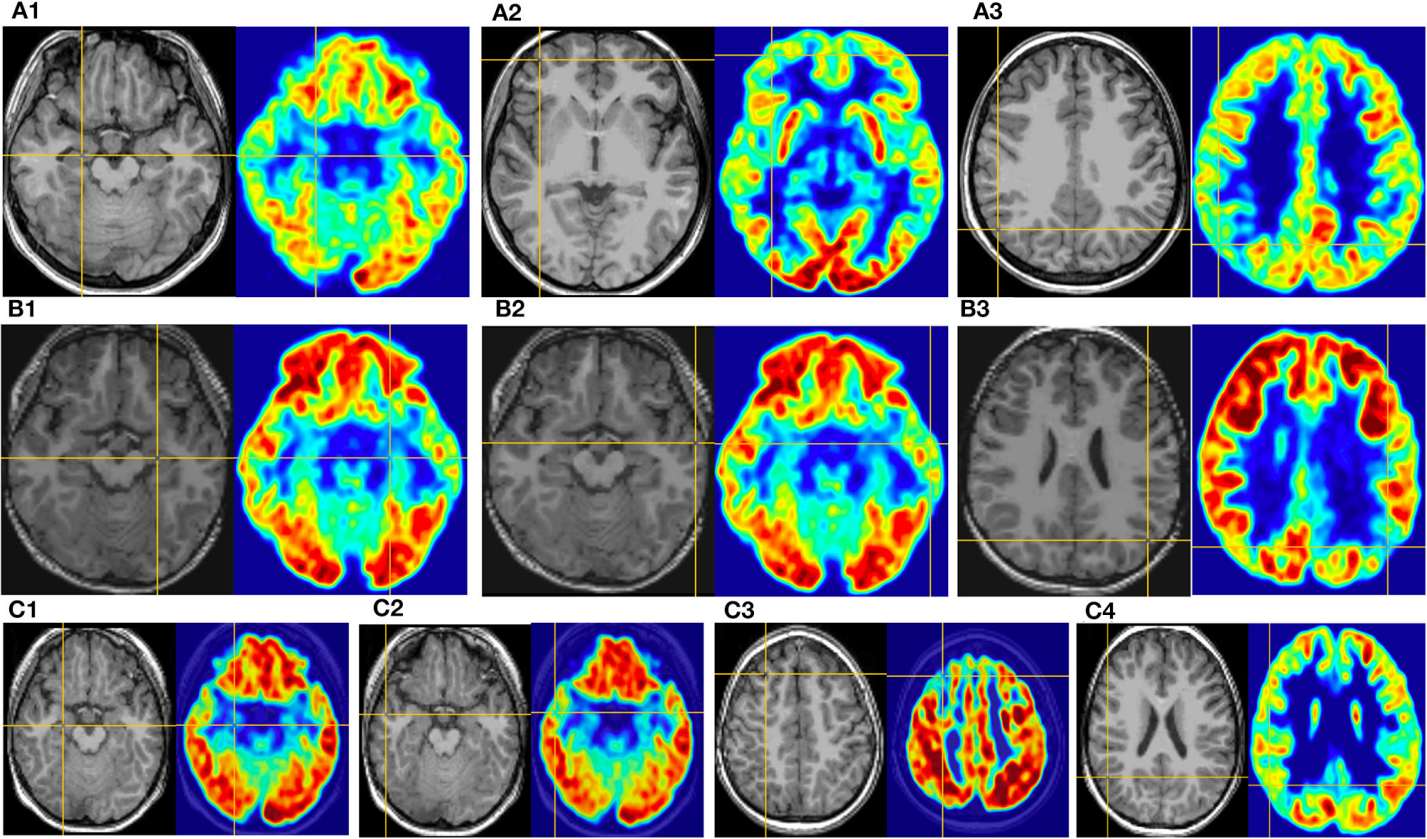
This is the MRI-PET of Pt. 16 **(A)**, Pt. 9 **(B)**, and Pt. 5 **(C)** in the third cortical hypometabolic pattern group. T1-MPRAGE (left) and PET images (right) were co-registered and normalized in MNI reference. **(A)** The cortical hypometabolic region is located in the right mesial temporal cortex **(A1)**, right frontal cortex **(A2)**, and right parietal cortex **(A3)**. **(B)** The cortical hypometabolic region is located in the left mesial temporal cortex **(B1)**, left lateral temporal cortex **(B2)**, and left parietal cortex **(B3)**. **(C)** The cortical hypometabolic region is located in the right mesial temporal cortex **(C1)**, right lateral temporal cortex **(C2)**, right frontal cortex **(C3)**, and right parietal cortex **(C4)**.

**Figure 6:**
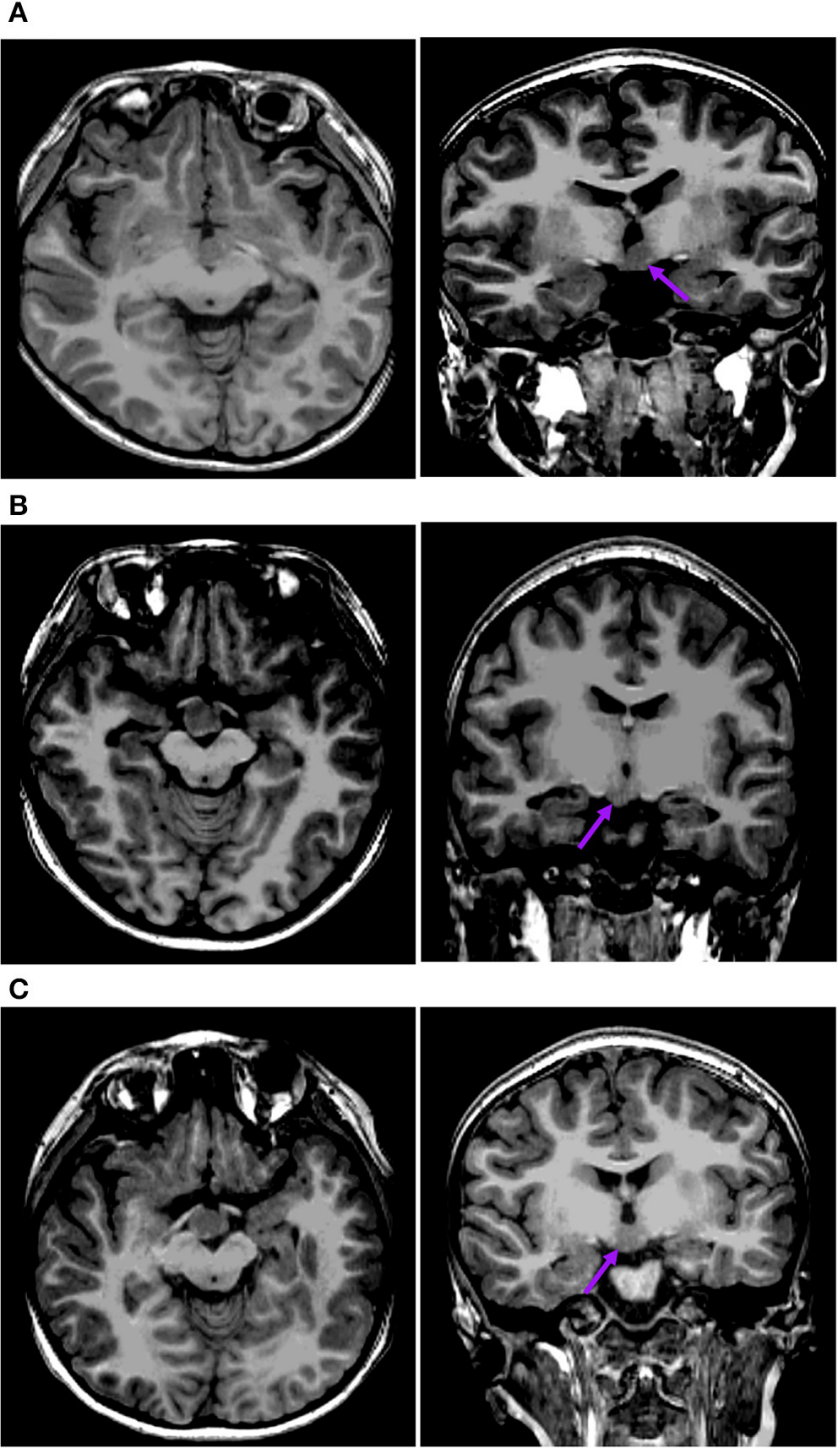
This is the raw T1-MPRAGE images of Pt. 9 **(A)**, Pt. 16 **(B)**, and Pt. 5 **(C)** in the third cortical hypometabolic pattern group. The T1-MPRAGE images (arrows) of **(A–C)** showed HH masses extended both above and below the third ventricle. The upper portion of the HH mass was located in the third ventricle, which closely connected with the middle nucleus of the hypothalamus. The lower portion was located below the third ventricle, tightly connected with the mammillary bodies.

The third pattern (pattern III) among the other eight patients showed the most extensive range of reduced glucose metabolism in the extra-hypothalamic cortex, including the mesial and lateral temporal, frontal, and parietal cortex (Figure 5). Two patients (Pt. 3 and 16) presented cortical hypometabolism in the mesial temporal cortex, frontal cortex, and parietal cortex (Figure 5A). Two patients (Pt. 2 and 9) presented cortical hypometabolism in the mesial temporal cortex, the lateral temporal cortex, and the parietal cortex (Figure 5B). Four patients (Pt. 5, 6, 8, and 12) presented cortical hypometabolism in the mesial temporal cortex, the lateral temporal cortex, the frontal cortex, and the parietal cortex (Figure 5C). The T1-MPRAGE of the patients showed that HH masses extended both above and below the third ventricle. The upper portion of the HH mass was located in the third ventricle, which closely connected with the middle nucleus of the hypothalamus. The lower portion was located below the third ventricle, tightly connected with the mammillary bodies (Figure 6). Of the eight patients with the third hypometabolic pattern, the interictal EEG discharge showed temporal spike (one of eight), temporal-parietal spike (one of eight), or diffused spike (six of eight). The ictal EEG discharge showed diffused electrodecremental events or diffused paroxysmal fast activity. The seizure types in this group included gelastic seizure (eight of eight), focal impaired awareness seizure (six of eight), and focal to generalized tonic-clonic seizure (four of eight).

### Voxel-Wise FDG-PET Analyses of ROI in Temporal Cortex

Table 3 presents the mean normalized metabolic voxel values from PET. In the first hypometabolic pattern group, the three patients (Pt. 4, 7, and 10) showed >10% asymmetry value in hippocampal formation. No patient showed asymmetry in the amygdala and the lateral temporal neocortex. In the second hypometabolic pattern group, five patients (Pt. 1, 11, 13, 14, and 15) showed >10% asymmetry value in the lateral temporal neocortex, but no patients showed >10% asymmetry value in the hippocampal formation and amygdala. In the third hypometabolic pattern group, >10% asymmetry value was found in eight patients within the hippocampal formation, four patients (Pt. 5, 8, 9, and 16) within the amygdala, and five patients (Pt. 2, 6, 8, 9, and 12) within the lateral temporal neocortex.

**Table 3:** Quantitative analysis of [18F] FDG PET of ROI in temporal cortex.

**No**.	**Con.HF**	**Ips.HF**	**MAV**	**Con.Amy**	**Ips.Amy**	**MAV**	**Con.Lat_TEN**	**Ips.Lat_TEN**	**MAV**
1	1.718	1.569	0.095	0.837	0.776	0.079	3.452	3.085	0.119^a^
2	2.101	1.825	0.151^a^	0.824	0.793	0.039	4.740	3.478	0.363^a^
3	1.826	1.613	0.132^a^	0.869	0.859	0.011	3.940	3.741	0.053
4	2.103	1.896	0.109^a^	1.068	1.005	0.063	3.622	3.557	0.018
5	1.873	1.696	0.105^a^	0.870	0.789	0.103^a^	4.393	4.216	0.042
6	2.175	1.943	0.119^a^	0.905	0.901	0.005	4.226	3.811	0.109^a^
7	1.962	1.759	0.115^a^	0.941	0.881	0.069	4.322	4.000	0.080
8	2.106	1.810	0.163^a^	0.999	0.889	0.124^a^	3.756	3.190	0.177^a^
9	2.013	1.821	0.106^a^	0.965	0.871	0.107^a^	4.066	3.681	0.105^a^
10	1.970	1.771	0.113^a^	0.783	0.729	0.074	3.918	3.720	0.053
11	2.200	2.125	0.035	0.953	0.944	0.009	4.751	4.274	0.112^a^
12	1.970	1.766	0.115^a^	0.971	0.873	0.039	4.158	3.705	0.122^a^
13	1.646	1.597	0.031	0.838	0.828	0.012	3.450	3.116	0.107^a^
14	1.755	1.673	0.049	0.928	0.830	0.099	3.952	3.431	0.152^a^
15	2.163	2.141	0.011	1.048	0.962	0.090	4.285	3.809	0.125^a^
16	1.909	1.720	0.110^a^	0.876	0.787	0.113^a^	3.862	3.662	0.054

*Con, contralateral; Ips ipsilateral; HF, hippocampal formation; MAV, metabolism asymmetric value; Amy, amygdala; Lat_TEN, lateral temporal neocortex, a > 10%*.

**Figure 7:**
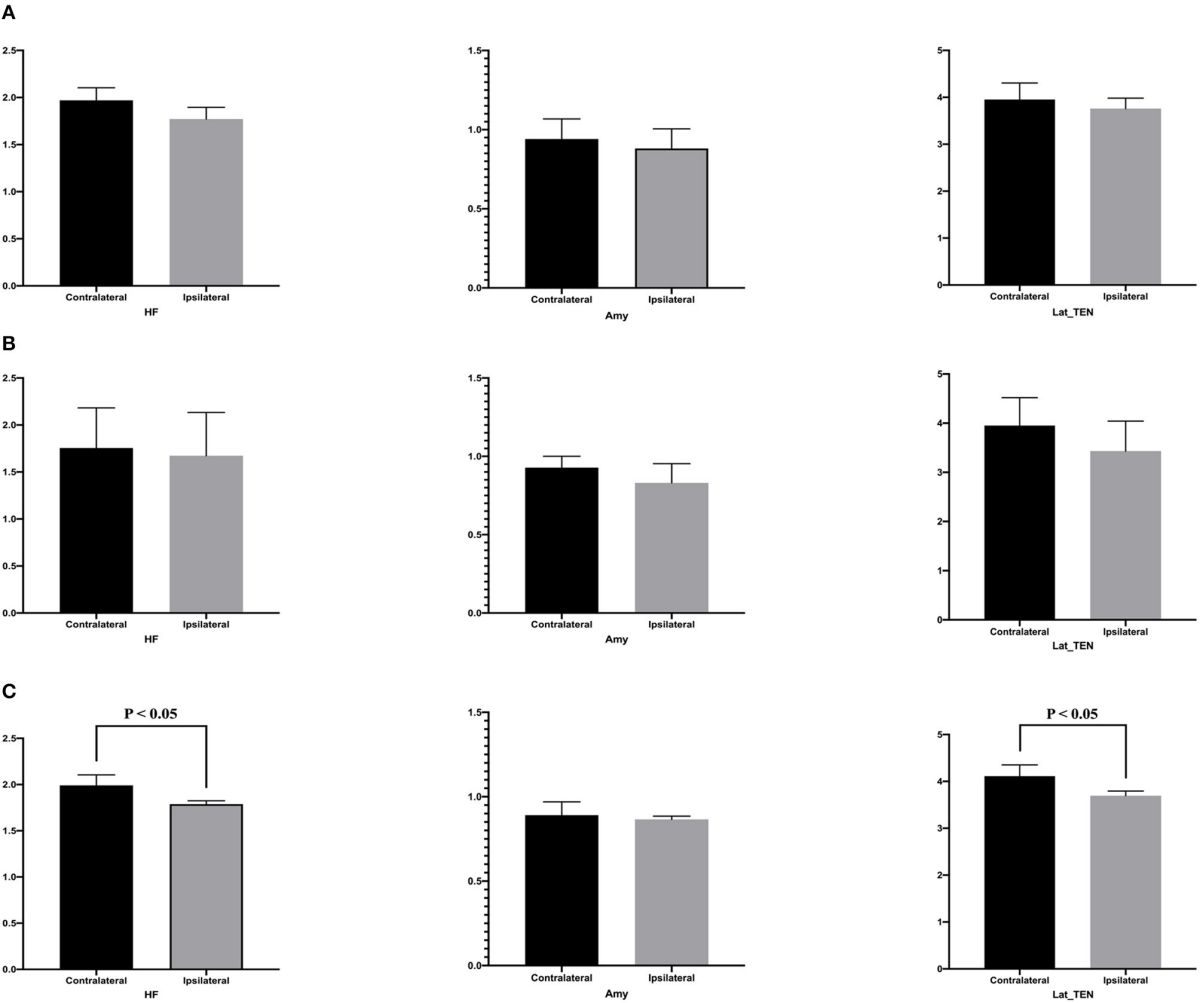
This is the quantitative analysis of asymmetry measurements of [^18^F] FDG PET in ROI of the temporal cortex. No statistical asymmetry measurements were found between the hippocampal formation, the amygdala, and the lateral temporal neocortex, in the first **(A)** and second groups **(B)**. **(C)** In the pattern III hypometabolic pattern group, the mean normalized PET voxel values were significantly higher in the contralateral hippocampus formations than that in the ipsilateral hippocampus formations (median [IQR]: 1.992 [1.882–2.105] vs. 1.788 [1.702–1.824], *P* < 0.05); and the mean normalized PET voxel values were significantly higher in the contralateral lateral temporal neocortex than that in the ipsilateral lateral temporal neocortex (median [IQR]: 4.003 [3.783–4.334] vs. 3.672 [3.262–3.732], *P* < 0.05). No significant differences were found between bilateral amygdala.

Table 4 presents quantitative analysis of asymmetry value of [^18^F] FDG PET in ROI of the temporal cortex. In the hypometabolic pattern group I, no statistically significant asymmetry was found between the hippocampal formation (median [IQR]: 1.970 [1.909–2.103] vs. 1.771 [1.720–1.825], *P* = 0.100), amygdala (median [IQR]: 0.941 [0.869–0.971] vs. 0.881 [0.789–0.889], *P* = 0.700), and lateral temporal neocortex (median [IQR]: 3.918 [3.622–4.158] vs. 3.720 [3.478–3.741], *P* = 0.700). In the hypometabolic pattern group II, no statistically significant asymmetry was found between the hippocampal formation (median [IQR]: 1.755 [1.682–2.181] vs. 1.673 [1.583–2.133], *P* = 0.310), amygdala (median [IQR]: 0.928 [0.838–1.001] vs. 0.830 [0.802–0.953], *P* = 0.310), and lateral temporal neocortex (median [IQR]: 3.452 [3.451–4.518] vs. 3.431 [3.101–4.042], *P* = 0.222). In the hypometabolic pattern group III, the mean normalized PET voxel values were significantly higher in the contralateral hippocampus formations than that in the ipsilateral hippocampus formations (median [IQR]: 1.992 [1.882–2.105] vs. 1.788 [1.702–1.824], *P* = 0.001); and the mean normalized PET voxel values were significantly higher in the contralateral lateral temporal neocortex than that in the ipsilateral lateral temporal neocortex (median [IQR]: 4.003 [3.783–4.334] vs. 3.672 [3.262–3.732], *P* = 0.005). No significant differences were found between bilateral amygdale (median [IQR]: 0.891 [0.868–0.970] vs. 0.865 [0.790–0.885], *P* = 0.083) (Figure 7).

**Table 4:** Asymmetry measurements of PET voxel value of the temporal cortex of three hypometabolic patterns.

	**Con.HF**	**Ips.HF**	**Con.Amy**	**Ips.Amy**	**Con.Lat_TEN**	**Ips.Lat_TEN**
**PATTERN 1**						
Median [IQR]	1.970 [1.909–2.103]	1.771 [1.720–1.825]	0.941 [0.869–0.971]	0.881 [0.789–0.889]	3.918 [3.622–4.158]	3.720 [3.478–3.741]
*P*-value	0.100		0.700		0.700	
**PATTERN 2**						
Median [IQR]	1.755 [1.682–2.181]	1.673 [1.583–2.133]	0.928 [0.838–1.001]	0.830 [0.802–0.953]	3.452 [3.451–4.518]	3.431 [3.101–4.042]
*P*-value	0.310		0.310		0.222	
**PATTERN 3**						
Median [IQPR]	1.992 [1.882–2.105]	1.788 [1.702–1.824]	0.891 [0.868–0.970]	0.865 [0.790–0.885]	4.112 [3.881–4.351]	3.693 [3.524–3.794]
*P*-value	0.001		0.083		0.005	

*Con, contralateral; Ips, ipsilateral; HF, hippocampal formation; Amy, amygdala; Lat_TEN, lateral; IQR, interquartile range*.

### Surgical Prognosis

The prognosis after surgery of the hypometabolic pattern groups was defined according to the ILAE classification system. A good prognosis corresponded to ILAE classes 1, 2, and 3 (31). Of the first hypometabolic pattern I, there were two cases (2/3) of ILAE class 1 and one case (1/3) of class 4. For the pattern II, there were two cases of ILAE class 1 (2/5), two cases of class 2 (2/5), and one case of class 4 (1/5). In the pattern III, there were two cases of ILAE class 1 (2/8), one case of class 3 (1/8), four cases of class 4 (4/8), and one case of class 5 (1/8). Hence, the distribution of cases with good prognosis (classes 1–3) was as follows: pattern I (2/3, 66.7%), pattern II (4/5, 80%), and pattern III (3/8, 37.5%). For details, see Table 1.

## Discussion

To our knowledge, this is one study with a relatively large sample size focusing on metabolic distribution characteristics of extra-hypothalamic cortex in patients with HH. With respect to the cortical glucose metabolic characteristics in patients with HH, we found that HH mass lateralization seen on MRI was significantly correlated with the lateralization of cortical hypometabolism. This is consistent with previous findings. For example, Wagner et al.'s (6) cortical hypometabolism was on the ipsilateral side as the HH mass in their series. Two independent case reports of PET for HH showed temporal cortex hypometabolism ipsilateral to HH mass, consistent with the findings of the scalp and intracranial EEG (7, 32).

In addition, our research found that the hypometabolism pattern of extra-hypothalamic cortex can be divided into three patterns, mainly related to the neuroanatomical location of the HH mass and was concordant with the cortical regions of the interictal and ictal discharges. In a PET study of five patients with HH, Ryvlin et al. (21) found that the cortical hypometabolic patterns varied greatly among different patients, much like electroclinical findings. These hypometabolic cortexes include the temporal cortex, frontal cortex, and parietal cortex. The findings were consistent with our conclusions. However, Ryvlin's paper may be limited by the insufficient number of cases, and it was impossible to make a specific analysis of the correlation between the hypometabolic distribution pattern of the extra-hypothalamus cortex and the neuroanatomic characteristics of the HH mass. Previous studies have found that 75% of patients with HH can develop focal epilepsy with temporal or frontal lobe seizures, depending on the neuroanatomical connection of the HH mass (23, 24). In those studies, cases showing temporal lobe seizures may reportedly be related to the HH mass invasion of the mammillary bodies, while cases showing frontal lobe seizures may be tightly connected with the middle hypothalamic nuclei (25). Therefore, our paper investigated the correlation between the hypometabolic distribution pattern of extra-hypothalamic cortex and the characteristics of HH neuroanatomy and electrophysiology.

We found that the first pattern of cortical hypometabolism was in the limbic structures of the mesial temporal lobe. In this group of cases, MRI showed that HH masses were entirely located below the third ventricle and inside the interpeduncular cistern. The T1 image showed the structure of the mammillary bodies was interrupted and unclear, suggesting that HH masses may invade the mammillary bodies. Previous literature shows that HH have a tight connection with the limbic system, such as mesial temporal cortex, through the mammillary bodies (33). List et al. (34) found in the course of autopsy in a patient with HH, a large HH mass involved with the left mammillary body and showed clinical and EEG characteristics of temporal lobe seizures. Studies have confirmed that the mammillothalamic tract is the most prominent projection of hypothalamus to the cortex (35). The mammillothalamic tract first projected through the mammillary bodies to the anterior nucleus of the thalamus, then to the cingulate gyrus, finally completing the circuit of Papez by projecting back to the mesial temporal cortex. Electrophysiological studies have also confirmed this indirectly (25). In a group of patients with HH mass connected to the mammillary bodies, the scalp EEG showed a spike in the temporal lobe, especially the mesial temporal cortex. The spike activity might propagate to the subiculum through the fornix, then spread to the mesial temporal cortex throughout the commissural fibers. This may explain the restricted cortical hypometabolism in such patients. The EEG in our group was also mainly manifested as temporal spike, with sphenoid electrode predominantly. The main symptomology included gelastic seizure and focal-impaired awareness seizure. These EEG and clinical manifestation are also consistent with previous results, suggesting that the HH-related seizure can propagate to the mesial temporal cortex. Therefore, these evidences indicate that HH mass involving mammillary bodies may establish a wide range of anatomical and functional connections to the limbic structures such as the mesial temporal cortex through the mammillothalamic tract, eventually leading to hypometabolism in those regions.

Cortical hypometabolism in the second group of cases was mainly in the lateral temporal cortex, and it can also involve the extratemporal cortex, such as the frontal and parietal cortex. Consistently, MRI in this group showed HH masses mainly located above the third ventricle, inside the middle hypothalamic nucleus. The HH masses in this group were mainly attached to the middle nucleus of the hypothalamus, sparing the mammillary bodies. T1 images show the intact mammillary body structure without interruption. Therefore, the hypometabolism of the cortex in this group may be due to the anatomical and functional connection of the HH masses with the cortex through the middle hypothalamic nucleus. Anterograde tracing studies showed that the middle hypothalamic neurons project through the medial forebrain bundle into the cerebral cortex. At the preoptic area, fibers run ventrally to globus pallidus, putamen, and external capsule, from which they project to much of the lateral temporal cortex outside the hippocampal formation (35). A DTI study also identified that hypothalamic neurons also connected to the parietal and occipital regions through the temporooccipital fascicle (36). Connections to the frontal cortex were also found (6). The EEG in our group was mainly manifested as frontotemporal or temporal-parietal discharge. The main symptomology included gelastic seizure and focal to generalized tonic-clonic seizures. This is grossly concordant with previous studies, suggesting the HH mass involving the middle hypothalamic nucleus may have connections to the lateral temporal cortex and/or extratemporal (frontal or parietal) cortex, leading to hypometabolism in those regions.

The third group of cases has the widest range of cortical hypometabolic regions, including the mesial and lateral temporal cortex, frontal cortex, and parietal cortex. T1-MPRAGE of this group showed that the HH mass located both above and below the third ventricle, with part of the HH mass inside the third ventricle and with part across the floor of the third ventricle, entering the interpeduncular cisterna. The portion of the HH mass below the third ventricle may affect the mammillary bodies, while the portion of the HH mass above the third ventricle may involve the middle nucleus of the hypothalamus. The HH mass in this group can connect to extra-hypothalamic cortex through both pathways described in the first and second groups. Therefore, the cortical hypometabolic region of this group was the most extensive, and the cortex of EEG discharge was diffused, with weak localization values. The patient's seizure patterns were the most diverse, with gelastic seizure, focal impaired awareness seizure, and focal to generalized tonic-clonic seizures. Generalized tonic-clonic seizures showed the highest frequency in this group of patients. The prognosis of patients with the third type was the worst, which is also consistent with previous literature reports indicating that the higher the extension of the hypometabolism in cortex, the worst is the prognosis after surgery. The previous study showed the prognosis of those with hypometabolism in the mesial temporal lobe is better than that of those with hypometabolism in cortex other than the mesial temporal lobe among patients with mesial temporal epilepsy (37). This point also shows that the study of PET in HH patients is of great significance in guiding the preoperative evaluation of HH patients and judging the prognosis.

We believe that our study was an initial step toward metabolic characterization of patients with HH according to the clinical question that the single epileptogenic hamartoma might lead to cortical abnormality outside HH during the disease process. In our patients, the fact that the lateralization of hypometabolism always occurred ipsilateral in MRI and EEG findings reinforced the view that the ipsilateral extra-hypothalamic cortical area is affected during the disease process. As for the hypometabolism pattern of extra-hypothalamic cortex, we combined visual analysis with quantification of the asymmetry value because of the difficulty to obtain normative FDG data from matched healthy control subjects to compare with the affected patients, especially for children. The results demonstrated that, in the third group, differences were found between the contralateral and ispilateral sides of the hippocampal formation and the lateral temporal neocortex. By contrast, no statistically significant asymmetry was found between the hippocampal formations, or in the lateral temporal neocortex, in the first and second groups, respectively. Lack of asymmetry value in these groups may be a result of an insufficient sample size because significantly hypometabolic regions were present with visual rating in these patients. In the future, we will continue to enroll more cases to better characterize glucose metabolism associated with HH neuroanatomy and therein further elucidate the complex pathophysiology of aberrant connection with remote cortex in patients with HH.

Several limitations of our study must be pointed out. First, because PET is an invasive examination, PET data from a group of healthy individuals was difficult to be obtained, and there were no such data retained in the early stage. The asymmetry assessment method used in the paper ignored potential age-related changes in normal metabolic asymmetries and could not address potential bilateral hypometabolism in these regions. Second, EEG was not monitored during the tracer uptake period; thus, subclinical seizures or frequent interictal epileptiform activity might affect metabolism. Third, due to the small sample data and limited statistical analysis, the study was an initial step to suggest three hypometabolic patterns in patients with HH and the third pattern may be associated with poor prognosis; thus, more studies are needed to confirm these data in the future.

## Data Availability Statement

The original contributions presented in the study are included in the article/supplementary material, further inquiries can be directed to the corresponding author/s.

## Ethics Statement

The studies involving human participants were reviewed and approved by Ethic committee of Xuanwu Hospital of Capital Medical University, Beijing, China. Written informed consent to participate in this study was provided by the participants' legal guardian/next of kin. Written informed consent was obtained from the individual(s), and minor(s)' legal guardian/next of kin, for the publication of any potentially identifiable images or data included in this article.

## Author Contributions

Y-FY contributed to the data acquisition and analysis of the manuscript. Y-ZS and G-GZ contributed to the data acquisition, analysis and redaction of the manuscript, and also the interpretation of the data. P-HW, Y-HW, and L-KR contributed to the data acquisition and analysis. YA, FM, and DW contributed to data acquisition. All authors read and approved the final manuscript.

## Conflict of Interest

The authors declare that the research was conducted in the absence of any commercial or financial relationships that could be construed as a potential conflict of interest.

## Abbreviations

HH: hypothalamic hamartomas
[^18^F] FDG-PET: [^18^F]-fluorodesoxyglucose positron emission tomography
SPECT: single-photon emission computed tomography
EEG: electroencephalography
MRI: magnetic resonance imaging
MPRAGE: magnetization-prepared rapid-gradient echo
ROS: region of interest
IQR: interquartile range
ILAE: International League Against Epilepsy.
